# (*S*,*E*)-3-[(2-Hy­droxy­benzyl­idene)amino]-2-(2-hy­droxy­phen­yl)-2,3-dihydro­quinazolin-4(1*H*)-one

**DOI:** 10.1107/S1600536812030012

**Published:** 2012-07-07

**Authors:** Daniel Tinguiano, Adama Sy, Ibrahima Elhadj Thiam, Mohamed Gaye, Pascal Retailleau

**Affiliations:** a Département de Chimie, Faculté des Sciences et Techniques, Université Cheikh Anta Diop, Dakar, Senegal; b Centre de Recherche de Gif, Institut de Chimie des Substances Naturelles, CNRS-UPR2301, 1 Avenue la Terrasse, 91198 Gif sur Yvette, France

## Abstract

In the title compound, C_21_H_17_N_3_O_3_, the dihydro­quinazoline ring adopts a screw-boat conformation and its stereogenic C atom has an *S* configuration. The dihedral angle between the mean planes of the two hy­droxy­phenyl rings is 86.61 (12)°. The amino H atom forms an intra­molecular hydrogen bond with a phenol O atom, while the hydrazine N atom acts as an acceptor for the H atom of the other phenol group. In the crystal, O—H⋯N and O—H⋯O hydrogen bonds and weak C—H⋯centroid(π-ring) inter­molecular inter­actions are observed, forming chains along [1-10] and [110].

## Related literature

For related structures and their biological properties, see: Rádl *et al.* (2000 ▶); Andries *et al.* (2005 ▶); Alagarsamy *et al.* (2006 ▶); Ghorab *et al.* (2007 ▶); El-Azab *et al.* (2010 ▶). For puckering parameters, see: Cremer & Pople (1975 ▶). For determination of the absolute configuration, see: Flack (1983 ▶); Hooft *et al.* (2008 ▶).
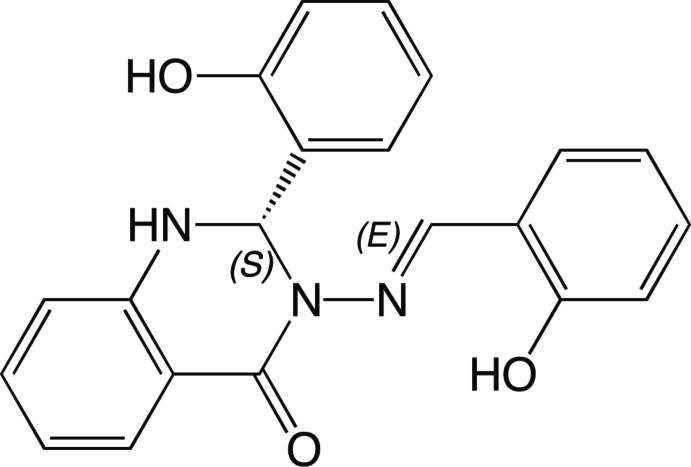


## Experimental

### 

#### Crystal data

C_21_H_17_N_3_O_3_
*M* *_r_* = 359.38
Orthorhombic, 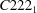

*a* = 13.344 (15) Å
*b* = 10.693 (14) Å
*c* = 23.537 (13) Å
*V* = 3358 (6) Å^3^
*Z* = 8
Cu *K*α radiation
μ = 0.79 mm^−1^
*T* = 193 K
0.41 × 0.34 × 0.16 mm

#### Data collection

Rigaku RAPID II R-AXIS conversion diffractometer
Absorption correction: multi-scan (*ABSCOR*; Higashi, 1995 ▶) *T*_min_ = 0.720, *T*_max_ = 0.889
15117 measured reflections
2947 independent reflections
2758 reflections with *I* > 2σ(*I*)
*R*_int_ = 0.039

#### Refinement

*R*[*F* ^2^ > 2σ(*F* ^2^)] = 0.035
*wR*(*F* ^2^) = 0.098
*S* = 1.07
2947 reflections
250 parameters
H atoms treated by a mixture of independent and constrained refinement
Δρ_max_ = 0.18 e Å^−3^
Δρ_min_ = −0.21 e Å^−3^
Absolute structure: Flack (1983 ▶), with 1258 Friedel pairs
Flack parameter: 0.0 (2)

### 

Data collection: *CrystalClear-SM Expert* (Rigaku, 2009 ▶); cell refinement: *CrystalClear-SM Expert*; data reduction: *CrystalClear-SM Expert*; program(s) used to solve structure: *SHELXS97* (Sheldrick, 2008 ▶); program(s) used to refine structure: *SHELXL97* (Sheldrick, 2008 ▶) interfaced by *CRYSTALBUILDER* (Welter, 2006 ▶); molecular graphics: *PLATON* (Spek, 2009 ▶) and *Mercury* (Macrae *et al.*, 2008 ▶); software used to prepare material for publication: *publCIF* (Westrip, 2010 ▶) and *PLATON*.

## Supplementary Material

Crystal structure: contains datablock(s) I, global. DOI: 10.1107/S1600536812030012/jj2144sup1.cif

Structure factors: contains datablock(s) I. DOI: 10.1107/S1600536812030012/jj2144Isup2.hkl

Supplementary material file. DOI: 10.1107/S1600536812030012/jj2144Isup3.cml

Additional supplementary materials:  crystallographic information; 3D view; checkCIF report

## Figures and Tables

**Table 1:** Hydrogen-bond geometry (Å, °) *Cg*3 is the centroid of the C16–C21 benzene ring.

*D*—H⋯*A*	*D*—H	H⋯*A*	*D*⋯*A*	*D*—H⋯*A*
O3—H3O⋯N3	0.84	1.95	2.704 (4)	148
O2—H2O⋯O1^i^	0.84	1.73	2.555 (3)	168
C4—H4⋯*Cg*3^ii^	0.93	2.64	3.546 (5)	160
C5—H5⋯*Cg*3^iii^	0.93	2.91	3.705 (5)	141

Symmetry codes: (i) 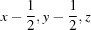; (ii) 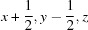; (iii) 
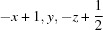
.

**Fig. 1.**
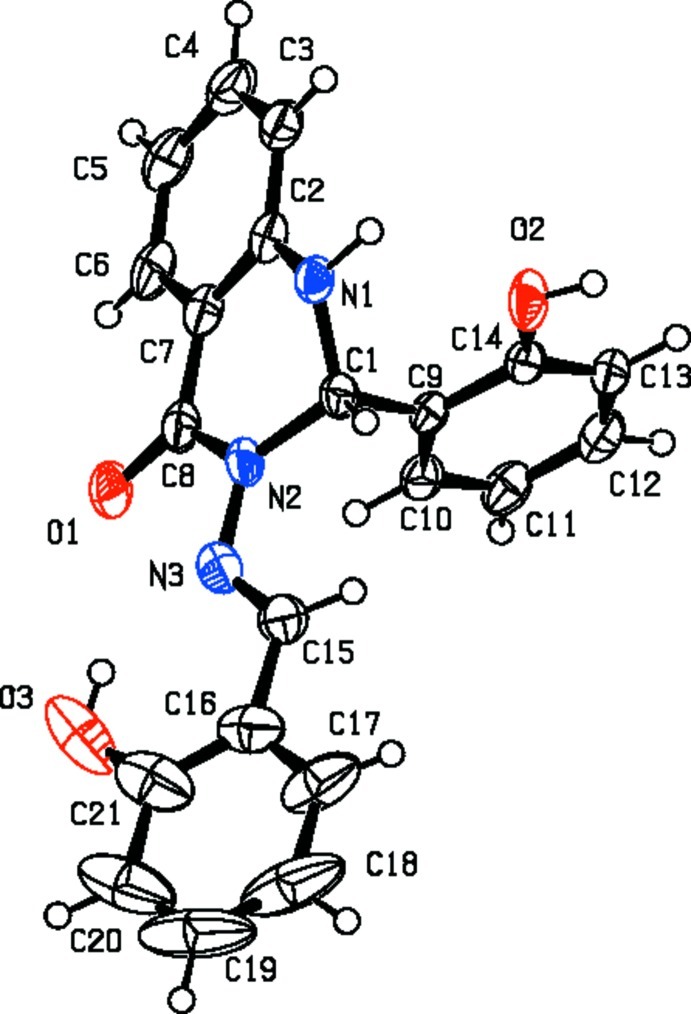
An ORTEP view of the title compound, showing the atom-numbering scheme. Displacement ellipsoids are plotted at the 50% probability level.

**Fig. 2.**
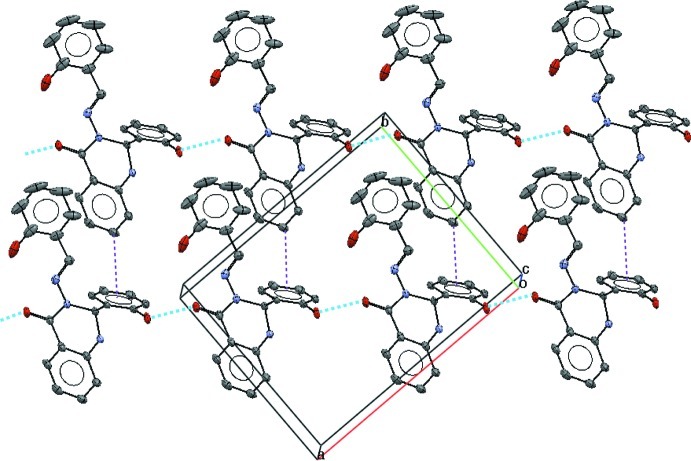
Molecular representation of the compound viewed along the c axis. Cyan dashed lines represent O—H···O hydrogen bonds and violet dashed lines indicate weak C—H···Cg π-ring intermolecular interactions.

## supplementary crystallographic information

### Comment

Quinazolinone derivatives give rise to a large spectrum of biological properties such as analgesic (Rádl *et al.*, 2000), antitubercular (Andries *et al.*, 2005), antibacterial (Alagarsamy *et al.*, 2006), anticancer activities (Ghorab *et al.*, 2007) and anti-convulsant activity (El-Azab *et al.*, 2010). We report here the crystal structure of the title compound, (I), and its absolute configuration. In the asymmetric unit of (I), the dihydroquinazolin ring adopts a screw-boat conformation with puckering parameters Q, θ, and φ of 0.4408 (18) Å, 64.1 (2)° and 41.9 (3)°, respectively (Cremer & Pople, 1975). The compound assumes an *E* configuration about the C═N double bond and its stereogenic C atom has an S configuration (Flack, 1983; Hooft *et al.*, 2008) (Fig. 1). The amino H atom forms an intramolecular hydrogen bond with a phenolic O atom while the hydrazino N acts as acceptor for the H atom of the other phenolic group. The dihedral angle between the 2-hydroxyphenyl rings is 86.61 (12)°. O—H···N and O—H···O hydrogen bonds and weak C—H···*Cg*π-ring intermolecular interactions are observed (Table 1) forming chains along [110] and [110] showing a corrugated layered structure in the *ab* plane which contributes to crystal packing stability (Fig. 2).

### Experimental

*o*-Aminobenzoylhydrazine (0.302 g, 2 mmol) was dissolved in 5 ml ethanol and salicylaldehyde (0.488 g, 4 mmol) was added with thorough shaking. The mixture was heated under reflux during 2 h. On cooling, crystals that separated from the yellow solution were filtered off and recrystallized in methanol. Crystals suitable for X-ray analysis were obtained after 2 d. Yield: 73.5%. Anal. Calc. for [C_21_H_17_N_3_O_3_] (%): C, 70.18; H, 4.77; N, 11.69. Found: C, 70.16; H, 4.75; N, 11.71.

### Refinement

All H atoms were located in difference maps. The hydroxyl ones and those attached to the C atoms were then treated as riding in geometrically idealized positions, with O—H = 0.82 (AFIX 147), C—H = 0.93 (aromatic), and 0.98 Å (aliphatic), and with *U*_iso_(H) = k*U*_eq_(C, O), where k = 1.2, and 1.5 for the O atoms. The amino H atom was freely refined except for the isotropic displacement parameter constrained to *U*_iso_(H) = 1.2*U*_eq_(N). The Flack parameter was *x* = 0.0 (2) (Flack, 1983). Further analysis of the absolute structure in absence of atoms heavier than oxygen was performed using likelihood methods (Hooft *et al.*, 2008) with *PLATON* (Spek, 2009). A total of 1258 Bijvoet pairs (coverage of 0.94) were included in the calculations. The resulting value of the Hooft parameter was *y* = -0.10 (8), with a P3 probability for an inverted structure smaller than 0.9 × 10^-43^. These results indicated that the absolute structure has been correctly assigned.

### Crystal data

**Table:**

C_21_H_17_N_3_O_3_	*F*(000) = 1504
*M**_r_* = 359.38	*D*_x_ = 1.422 Mg m^−^^3^
Orthorhombic, *C*222_1_	Cu *K*α radiation, λ = 1.54187 Å
Hall symbol: C 2c 2	Cell parameters from 7031 reflections
*a* = 13.344 (15) Å	θ = 1.9–68.2°
*b* = 10.693 (14) Å	µ = 0.79 mm^−^^1^
*c* = 23.537 (13) Å	*T* = 193 K
*V* = 3358 (6) Å^3^	Block, colourless
*Z* = 8	0.41 × 0.34 × 0.16 mm

### Data collection

**Table:**

Rigaku RAPID II R-AXIS conversion diffractometer	2947 independent reflections
Radiation source: fine-focus rotating anode	2758 reflections with *I* > 2σ(*I*)
Graphite monochromator	*R*_int_ = 0.039
profile data from ω scans	θ_max_ = 68.2°, θ_min_ = 3.8°
Absorption correction: multi-scan (*ABSCOR*; Higashi, 1995)	*h* = −16→16
*T*_min_ = 0.720, *T*_max_ = 0.889	*k* = −12→11
15117 measured reflections	*l* = −28→27

### Refinement

**Table:**

Refinement on *F*^2^	Hydrogen site location: difference Fourier map
Least-squares matrix: full	H atoms treated by a mixture of independent and constrained refinement
*R*[*F*^2^ > 2σ(*F*^2^)] = 0.035	*w* = 1/[σ^2^(*F*_o_^2^) + (0.056*P*)^2^ + 1.1889*P*] where *P* = (*F*_o_^2^ + 2*F*_c_^2^)/3
*wR*(*F*^2^) = 0.098	(Δ/σ)_max_ < 0.001
*S* = 1.07	Δρ_max_ = 0.18 e Å^−^^3^
2947 reflections	Δρ_min_ = −0.21 e Å^−^^3^
250 parameters	Extinction correction: *SHELXL97* (Sheldrick, 2008), Fc^*^=kFc[1+0.001xFc^2^λ^3^/sin(2θ)]^-1/4^
0 restraints	Extinction coefficient: 0.00069 (11)
Primary atom site location: structure-invariant direct methods	Absolute structure: Flack (1983), with 1258 Friedel pairs
Secondary atom site location: difference Fourier map	Flack parameter: 0.0 (2)

### Special details

**Table:**

Experimental. Selected IR data (cm^-1^, KBr pellet): 3400, 3216, 1730, 1650, 1582, 1458, 764.
Geometry. All e.s.d.'s (except the e.s.d. in the dihedral angle between two l.s. planes) are estimated using the full covariance matrix. The cell e.s.d.'s are taken into account individually in the estimation of e.s.d.'s in distances, angles and torsion angles; correlations between e.s.d.'s in cell parameters are only used when they are defined by crystal symmetry. An approximate (isotropic) treatment of cell e.s.d.'s is used for estimating e.s.d.'s involving l.s. planes.
Refinement. Refinement of *F*^2^ against ALL reflections. The weighted *R*-factor *wR* and goodness of fit *S* are based on *F*^2^, conventional *R*-factors *R* are based on *F*, with *F* set to zero for negative *F*^2^. The threshold expression of *F*^2^ > σ(*F*^2^) is used only for calculating *R*-factors(gt) *etc*. and is not relevant to the choice of reflections for refinement. *R*-factors based on *F*^2^ are statistically about twice as large as those based on *F*, and *R*-factors based on ALL data will be even larger.

### Fractional atomic coordinates and isotropic or equivalent isotropic displacement parameters (Å^2^)

**Table:**

	*x*	*y*	*z*	*U*_iso_*/*U*_eq_	
O1	0.49079 (10)	0.40376 (14)	0.35557 (7)	0.0471 (4)	
O2	0.15925 (9)	0.01452 (15)	0.36712 (6)	0.0432 (4)	
H2O	0.1100	−0.0300	0.3621	0.052*	
O3	0.3763 (2)	0.6657 (2)	0.43629 (9)	0.0793 (7)	
H3O	0.3861	0.5928	0.4270	0.119*	
N1	0.34660 (10)	0.08134 (15)	0.39518 (6)	0.0305 (3)	
H1N	0.3110 (16)	0.005 (2)	0.3973 (9)	0.037*	
N2	0.35606 (10)	0.31007 (15)	0.38330 (7)	0.0320 (4)	
N3	0.32933 (12)	0.43142 (16)	0.40226 (7)	0.0372 (4)	
C1	0.29276 (12)	0.19782 (16)	0.38113 (8)	0.0288 (4)	
H1	0.2401	0.2085	0.4098	0.035*	
C2	0.42664 (12)	0.06210 (18)	0.36295 (8)	0.0314 (4)	
C3	0.46168 (13)	−0.06173 (19)	0.35216 (9)	0.0382 (5)	
H3	0.4282	−0.1310	0.3667	0.046*	
C4	0.54310 (14)	−0.0748 (2)	0.32088 (9)	0.0458 (5)	
H4	0.5682	−0.1535	0.3120	0.055*	
C5	0.59031 (15)	0.0343 (2)	0.30160 (10)	0.0480 (6)	
H5	0.6475	0.0251	0.2794	0.058*	
C6	0.55737 (14)	0.1572 (2)	0.31343 (9)	0.0415 (5)	
H6	0.5928	0.2260	0.2999	0.050*	
C7	0.47477 (13)	0.17286 (19)	0.34431 (8)	0.0345 (4)	
C8	0.44402 (13)	0.30414 (18)	0.36033 (8)	0.0341 (4)	
C9	0.24182 (11)	0.18717 (17)	0.32475 (8)	0.0281 (4)	
C10	0.25851 (13)	0.26696 (18)	0.27833 (8)	0.0326 (4)	
H10	0.3038	0.3326	0.2821	0.039*	
C11	0.21035 (14)	0.2508 (2)	0.22803 (8)	0.0385 (5)	
H11	0.2223	0.3039	0.1975	0.046*	
C12	0.14367 (14)	0.1541 (2)	0.22371 (9)	0.0405 (5)	
H12	0.1100	0.1411	0.1896	0.049*	
C13	0.12505 (13)	0.0739 (2)	0.26992 (8)	0.0355 (4)	
H13	0.0793	0.0089	0.2660	0.043*	
C14	0.17329 (12)	0.09045 (18)	0.32014 (8)	0.0313 (4)	
C15	0.24436 (16)	0.45674 (19)	0.41379 (8)	0.0398 (5)	
H15	0.1938	0.3979	0.4086	0.048*	
C16	0.2200 (2)	0.5842 (2)	0.43671 (9)	0.0511 (6)	
C17	0.1267 (2)	0.6088 (3)	0.44981 (11)	0.0760 (10)	
H17	0.0772	0.5493	0.4430	0.091*	
C18	0.1004 (4)	0.7261 (5)	0.47435 (13)	0.1121 (18)	
H18	0.0330	0.7395	0.4825	0.135*	
C19	0.1639 (5)	0.8182 (4)	0.48665 (14)	0.119 (2)	
H19	0.1430	0.8919	0.5040	0.143*	
C20	0.2560 (4)	0.7988 (3)	0.47299 (11)	0.0920 (13)	
H20	0.3041	0.8603	0.4791	0.110*	
C21	0.2836 (3)	0.6810 (3)	0.44825 (10)	0.0665 (8)	

### Atomic displacement parameters (Å^2^)

**Table:**

	*U*^11^	*U*^22^	*U*^33^	*U*^12^	*U*^13^	*U*^23^
O1	0.0269 (6)	0.0451 (8)	0.0693 (10)	−0.0139 (6)	−0.0023 (6)	0.0087 (8)
O2	0.0262 (7)	0.0524 (9)	0.0510 (8)	−0.0173 (6)	−0.0047 (6)	0.0142 (7)
O3	0.1221 (19)	0.0582 (12)	0.0577 (12)	−0.0448 (12)	0.0036 (12)	−0.0071 (10)
N1	0.0215 (7)	0.0338 (9)	0.0362 (8)	−0.0038 (6)	−0.0006 (6)	0.0028 (7)
N2	0.0227 (7)	0.0335 (9)	0.0399 (8)	−0.0067 (6)	−0.0011 (6)	−0.0027 (7)
N3	0.0384 (9)	0.0357 (9)	0.0374 (8)	−0.0059 (7)	−0.0025 (7)	−0.0016 (7)
C1	0.0215 (7)	0.0305 (10)	0.0344 (9)	−0.0040 (7)	0.0027 (7)	0.0000 (8)
C2	0.0199 (8)	0.0429 (11)	0.0314 (9)	−0.0012 (7)	−0.0019 (7)	0.0001 (8)
C3	0.0260 (9)	0.0424 (11)	0.0461 (11)	−0.0003 (8)	−0.0039 (8)	0.0030 (10)
C4	0.0293 (9)	0.0570 (14)	0.0513 (12)	0.0113 (9)	−0.0021 (8)	−0.0075 (11)
C5	0.0242 (9)	0.0714 (16)	0.0483 (12)	0.0063 (9)	0.0037 (8)	0.0000 (12)
C6	0.0205 (9)	0.0598 (13)	0.0443 (12)	−0.0021 (8)	0.0015 (8)	0.0080 (10)
C7	0.0223 (8)	0.0452 (11)	0.0360 (10)	−0.0027 (8)	−0.0042 (7)	0.0052 (9)
C8	0.0239 (8)	0.0406 (11)	0.0378 (10)	−0.0070 (8)	−0.0051 (7)	0.0051 (9)
C9	0.0164 (7)	0.0310 (9)	0.0370 (9)	0.0018 (6)	0.0015 (7)	−0.0023 (8)
C10	0.0217 (8)	0.0348 (10)	0.0413 (10)	0.0003 (7)	0.0035 (7)	0.0021 (8)
C11	0.0283 (8)	0.0512 (12)	0.0360 (10)	0.0040 (9)	0.0030 (8)	0.0087 (9)
C12	0.0287 (9)	0.0524 (13)	0.0405 (10)	0.0083 (9)	−0.0070 (8)	−0.0051 (9)
C13	0.0229 (8)	0.0360 (10)	0.0477 (11)	0.0001 (7)	−0.0068 (8)	−0.0056 (9)
C14	0.0212 (8)	0.0309 (10)	0.0416 (10)	−0.0005 (7)	0.0009 (7)	0.0006 (8)
C15	0.0427 (11)	0.0393 (11)	0.0376 (10)	0.0027 (9)	−0.0060 (9)	−0.0039 (8)
C16	0.0797 (16)	0.0419 (13)	0.0317 (10)	0.0166 (12)	−0.0107 (10)	−0.0029 (9)
C17	0.088 (2)	0.093 (2)	0.0472 (14)	0.0530 (18)	−0.0220 (14)	−0.0189 (14)
C18	0.158 (4)	0.125 (3)	0.0533 (17)	0.105 (3)	−0.035 (2)	−0.033 (2)
C19	0.239 (6)	0.072 (3)	0.0467 (18)	0.086 (3)	−0.030 (3)	−0.0183 (17)
C20	0.198 (4)	0.0386 (17)	0.0398 (14)	0.003 (2)	−0.017 (2)	−0.0004 (12)
C21	0.120 (3)	0.0441 (15)	0.0353 (12)	−0.0033 (16)	−0.0030 (14)	0.0052 (10)

### Geometric parameters (Å, º)

**Table:**

O1—C8	1.240 (3)	C7—C8	1.510 (3)
O2—C14	1.385 (2)	C9—C14	1.385 (3)
O2—H2O	0.8200	C9—C10	1.404 (3)
O3—C21	1.280 (4)	C10—C11	1.358 (3)
O3—H3O	0.8200	C10—H10	0.9300
N1—C2	1.326 (3)	C11—C12	1.368 (3)
N1—C1	1.475 (3)	C11—H11	0.9300
N1—H1N	0.95 (2)	C12—C13	1.407 (3)
N2—C8	1.294 (3)	C12—H12	0.9300
N2—N3	1.418 (3)	C13—C14	1.358 (3)
N2—C1	1.469 (3)	C13—H13	0.9300
N3—C15	1.197 (3)	C15—C16	1.501 (3)
C1—C9	1.495 (3)	C15—H15	0.9300
C1—H1	0.9800	C16—C17	1.310 (4)
C2—C7	1.417 (3)	C16—C21	1.365 (4)
C2—C3	1.427 (3)	C17—C18	1.425 (5)
C3—C4	1.320 (3)	C17—H17	0.9300
C3—H3	0.9300	C18—C19	1.331 (7)
C4—C5	1.401 (4)	C18—H18	0.9300
C4—H4	0.9300	C19—C20	1.288 (6)
C5—C6	1.414 (4)	C19—H19	0.9300
C5—H5	0.9300	C20—C21	1.436 (5)
C6—C7	1.331 (3)	C20—H20	0.9300
C6—H6	0.9300		
			
C14—O2—H2O	109.5	C10—C9—C1	124.91 (16)
C21—O3—H3O	109.5	C11—C10—C9	121.75 (18)
C2—N1—C1	113.27 (15)	C11—C10—H10	119.1
C2—N1—H1N	107.5 (13)	C9—C10—H10	119.1
C1—N1—H1N	119.7 (12)	C10—C11—C12	117.93 (19)
C8—N2—N3	113.87 (15)	C10—C11—H11	121.0
C8—N2—C1	117.86 (16)	C12—C11—H11	121.0
N3—N2—C1	127.89 (15)	C11—C12—C13	121.21 (18)
C15—N3—N2	121.12 (17)	C11—C12—H12	119.4
N2—C1—N1	113.71 (16)	C13—C12—H12	119.4
N2—C1—C9	110.76 (15)	C14—C13—C12	120.64 (18)
N1—C1—C9	110.85 (14)	C14—C13—H13	119.7
N2—C1—H1	107.1	C12—C13—H13	119.7
N1—C1—H1	107.1	C13—C14—O2	123.69 (17)
C9—C1—H1	107.1	C13—C14—C9	118.61 (18)
N1—C2—C7	114.36 (18)	O2—C14—C9	117.69 (16)
N1—C2—C3	120.64 (17)	N3—C15—C16	119.5 (2)
C7—C2—C3	124.88 (17)	N3—C15—H15	120.3
C4—C3—C2	117.8 (2)	C16—C15—H15	120.3
C4—C3—H3	121.1	C17—C16—C21	113.1 (3)
C2—C3—H3	121.1	C17—C16—C15	118.2 (3)
C3—C4—C5	117.6 (2)	C21—C16—C15	128.7 (3)
C3—C4—H4	121.2	C16—C17—C18	120.4 (4)
C5—C4—H4	121.2	C16—C17—H17	119.8
C4—C5—C6	124.8 (2)	C18—C17—H17	119.8
C4—C5—H5	117.6	C19—C18—C17	125.6 (4)
C6—C5—H5	117.6	C19—C18—H18	117.2
C7—C6—C5	118.8 (2)	C17—C18—H18	117.2
C7—C6—H6	120.6	C20—C19—C18	115.8 (3)
C5—C6—H6	120.6	C20—C19—H19	122.1
C6—C7—C2	116.1 (2)	C18—C19—H19	122.1
C6—C7—C8	118.56 (18)	C19—C20—C21	119.1 (4)
C2—C7—C8	125.20 (17)	C19—C20—H20	120.4
O1—C8—N2	116.90 (19)	C21—C20—H20	120.4
O1—C8—C7	129.74 (18)	O3—C21—C16	117.4 (3)
N2—C8—C7	113.35 (16)	O3—C21—C20	116.6 (3)
C14—C9—C10	119.84 (17)	C16—C21—C20	125.9 (4)
C14—C9—C1	115.25 (16)		
			
C8—N2—N3—C15	164.97 (18)	N2—C1—C9—C14	−174.44 (14)
C1—N2—N3—C15	−7.7 (3)	N1—C1—C9—C14	58.4 (2)
C8—N2—C1—N1	44.5 (2)	N2—C1—C9—C10	5.2 (2)
N3—N2—C1—N1	−143.08 (16)	N1—C1—C9—C10	−122.02 (19)
C8—N2—C1—C9	−81.11 (19)	C14—C9—C10—C11	−1.2 (3)
N3—N2—C1—C9	91.3 (2)	C1—C9—C10—C11	179.20 (17)
C2—N1—C1—N2	−55.6 (2)	C9—C10—C11—C12	0.4 (3)
C2—N1—C1—C9	69.98 (19)	C10—C11—C12—C13	0.3 (3)
C1—N1—C2—C7	31.0 (2)	C11—C12—C13—C14	−0.2 (3)
C1—N1—C2—C3	−152.58 (17)	C12—C13—C14—O2	−179.43 (16)
N1—C2—C3—C4	−178.41 (17)	C12—C13—C14—C9	−0.6 (3)
C7—C2—C3—C4	−2.4 (3)	C10—C9—C14—C13	1.3 (3)
C2—C3—C4—C5	1.3 (3)	C1—C9—C14—C13	−179.12 (16)
C3—C4—C5—C6	0.3 (3)	C10—C9—C14—O2	−179.83 (16)
C4—C5—C6—C7	−1.1 (3)	C1—C9—C14—O2	−0.2 (2)
C5—C6—C7—C2	0.1 (3)	N2—N3—C15—C16	176.71 (16)
C5—C6—C7—C8	175.72 (17)	N3—C15—C16—C17	−179.0 (2)
N1—C2—C7—C6	177.84 (17)	N3—C15—C16—C21	−1.5 (3)
C3—C2—C7—C6	1.6 (3)	C21—C16—C17—C18	−1.0 (4)
N1—C2—C7—C8	2.6 (3)	C15—C16—C17—C18	176.9 (2)
C3—C2—C7—C8	−173.65 (18)	C16—C17—C18—C19	−0.7 (5)
N3—N2—C8—O1	−3.1 (2)	C17—C18—C19—C20	2.5 (5)
C1—N2—C8—O1	170.36 (16)	C18—C19—C20—C21	−2.3 (5)
N3—N2—C8—C7	175.68 (14)	C17—C16—C21—O3	−179.4 (2)
C1—N2—C8—C7	−10.8 (2)	C15—C16—C21—O3	3.1 (4)
C6—C7—C8—O1	−10.5 (3)	C17—C16—C21—C20	1.0 (4)
C2—C7—C8—O1	164.6 (2)	C15—C16—C21—C20	−176.6 (2)
C6—C7—C8—N2	170.89 (17)	C19—C20—C21—O3	−178.9 (3)
C2—C7—C8—N2	−14.0 (3)	C19—C20—C21—C16	0.7 (4)

### Hydrogen-bond geometry (Å, º)

Cg3 is the centroid of which? ring.

**Table:**

*D*—H···*A*	*D*—H	H···*A*	*D*···*A*	*D*—H···*A*
O3—H3*O*···N3	0.84	1.95	2.704 (4)	148
O2—H2*O*···O1^i^	0.84	1.73	2.555 (3)	168
C4—H4···*Cg*3^ii^	0.93	2.64	3.546 (5)	160
C5—H5···*Cg*3^iii^	0.93	2.91	3.705 (5)	141

Symmetry codes: (i) *x*−1/2, *y*−1/2, *z*; (ii) *x*+1/2, *y*−1/2, *z*; (iii) −*x*+1, *y*, −*z*+1/2.
